# Oxidative Stress Responses and NRF2 in Human Leukaemia

**DOI:** 10.1155/2015/454659

**Published:** 2015-03-30

**Authors:** Amina Abdul-Aziz, David J. MacEwan, Kristian M. Bowles, Stuart A. Rushworth

**Affiliations:** ^1^Norwich Medical School, University of East Anglia, Norwich Research Park, Norwich NR4 7UQ, UK; ^2^Department of Molecular & Clinical Pharmacology, Institute of Translational Medicine, University of Liverpool, Liverpool L69 3GE, UK; ^3^Department of Haematology, Norfolk and Norwich University Hospitals National Health Service Trust, Norwich NR4 7UY, UK

## Abstract

Oxidative stress as a result of elevated levels of reactive oxygen species (ROS) has been observed in almost all cancers, including leukaemia, where they contribute to disease development and progression. However, cancer cells also express increased levels of antioxidant proteins which detoxify ROS. This includes glutathione, the major antioxidant in human cells, which has recently been identified to have dysregulated metabolism in human leukaemia. This suggests that critical balance of intracellular ROS levels is required for cancer cell function, growth, and survival. Nuclear factor (erythroid-derived 2)-like 2 (NRF2) transcription factor plays a dual role in cancer. Primarily, NRF2 is a transcription factor functioning to protect nonmalignant cells from malignant transformation and oxidative stress through transcriptional activation of detoxifying and antioxidant enzymes. However, once malignant transformation has occurred within a cell, NRF2 functions to protect the tumour from oxidative stress and chemotherapy-induced cytotoxicity. Moreover, inhibition of the NRF2 oxidative stress pathway in leukaemia cells renders them more sensitive to cytotoxic chemotherapy. Our improved understanding of NRF2 biology in human leukaemia may permit mechanisms by which we could potentially improve future cancer therapies. This review highlights the mechanisms by which leukaemic cells exploit the NRF2/ROS response to promote their growth and survival.

## 1. Introduction

Acute myeloid leukaemia (AML) is primarily a disease of the elderly with 75% of cases being diagnosed in patients over 60 years of age [1]. AML comprises a biologically heterogeneous group of disorders that occur as a consequence of a wide variety of genetic abnormalities in haematopoietic progenitors that are derived from the bone marrow. In fitter, generally younger patients complete remission can be achieved only in a minority with current chemotherapeutic regimens. Patients who are not fit for intensive chemotherapy are generally managed with a palliative approach without a chance of cure. Furthermore, even in patients who do achieve remission following intensive chemotherapy many relapse from the persistence of a small clone of minimal residual disease [2, 3] and, despite considerable efforts over the last 30 years to develop and improve therapy, presently two-thirds of younger adults and 90% of older adults still die of their disease [4]. It is envisaged that improved outcomes for all patients will now only come from novel treatment strategies (beyond increasing doses of conventional cytotoxic drugs) derived from an improved understanding of the biology of the disease.

## 2. Oxidative Stress

Oxidative stress is described as a change in the balance between reactive oxygen species and antioxidant defence mechanisms, where the balance is disturbed for the support of the oxidants [5]. Together, oxidants and antioxidants are essential for normal cellular function including metabolism and signal transduction which allow for the maintenance of cellular homeostasis [6, 7]. However, oxidative stress, if unconstrained, results in the damage of important cellular components which may result in DNA mutations or cell death.

Reactive oxygen species (ROS) are oxygen-containing chemical species with reactive properties, including free radicals such as superoxide and nonradical molecules such as hydrogen peroxide [8]. These reactive species result from both endogenous and exogenous cellular sources. Endogenous sources of cellular ROS include oxidative phosphorylation within mitochondria, which results in the formation of dioxygen, which is normally reduced to water but in some instances is partially reduced to form superoxide. Further reduction reactions can subsequently give rise to hydrogen peroxide [9, 10], which has long been thought of as a harmful molecule; however, recently, new evidence has emerged which suggests that at low concentrations hydrogen peroxide acts as an intracellular signaling molecule involved in survival and proliferation mechanism. In contrast, exogenous ROS is produced by many environmental mediators which have demonstrated involvement in a number of pathological states including cardiovascular disease [11], chronic inflammation [12], and neurodegenerative diseases [13] as well as cancer [14].

## 3. Reactive Oxygen Species

There is a complex interaction between ROS generation, signaling, and toxicity that results in the initiation, growth, and survival of cancer. Cancer may be induced through oxidative damage to cellular macromolecules as a result of overproduction of ROS, which subsequently affects antioxidant and/or DNA repair mechanisms [15]. In addition, ROS can stimulate signal transduction pathways leading to activation of key protumoural transcription factors [16]. Once the malignant state has been established, the same cellular survival mechanisms that the cell had employed to protect against tumorigenesis are subsequently subverted to support a protumoural state and protect the cancer cells from chemotherapy. ROS have a physiological cellular response to trigger cellular inflammation and damage that may lead to cell death. This protective effect is lost in cancer cells and thus endogenous and exogenous efforts to induce cytotoxicity may also be lost in cancer. Specifically in human leukaemia the NRF2 pathway appears central to the control of the redox state functioning at least in part through its regulation of glutathione synthesis and regeneration. It is envisaged that the identification of tumour-specific dependence within this pathway may ultimately be exploited to develop much needed new treatments.

## 4. Acute Myeloid Leukaemia

AML develops from a common myeloid progenitor, a cell which would physiologically differentiate to form monocytes, granulocytes, platelets, and erythrocytes in the bone marrow [17, 18]. AML is the most common acute leukaemia affecting adults, and its incidence increases with age [19]. However, AML is a heterogeneous disease driven by a wide variety of genetic lesions [20]. In patients fit enough for conventional intensive cytotoxic chemotherapy, the treatment destroys actively cycling leukaemic cells and initial remission rates are high. However, in these patients following remission induction and despite in many cases the disease becoming undetectable by current testing technologies, a subpopulation of cells with leukaemic stem cell properties frequently survives chemotherapy and it is this subpopulation (minimal residual disease) that is responsible for the relapse commonly encountered in this disease [21]. In patients not fit for such cytotoxic chemotherapy, management is presently based around palliation and symptom control.

The discovery of specific mutant genes in AML has provided increased biologic understanding, new potential targets for drug development [22], and new diagnostic methods for detection of minimal residual disease [23, 24]. For instance, mutations of the FMS-like tyrosine kinase-3 (FLT3) receptor (internal tandem duplication (ITD)) are found in approximately 25% of new cases of AML [25, 26]. FLT3-ITD has been found to cause increased levels of ROS within murine Ba/F3 or 32D cells expressing FlT3-ITD as well as MOLM-14 and MV-4-11 human AML cell lines which carry FLT3-ITD mutations [27], suggesting that ROS are important in regulating FLT3 mutated AML.

## 5. Manipulation of the Redox Status by Leukaemia Oncogenes

A number of oncogenes such as KRAS, cMYC, BCR/ABL, NRF2, and NF-kappaB (NF-*κ*B) are able to alter the redox balance of human cancer cells including leukaemic cells [26, 28–32]. The oncogenic BCR/ABL fusion gene found mainly chronic myeloid leukaemia (CML) is capable of inducing ROS levels in both human and murine cell lines [33, 34]. Moreover, BCR/ABL-induced ROS can also result in signaling changes including the upregulation of the nonreceptor tyrosine kinase FYN [35, 36]. FYN deficiency in the presence of BCR/ABL expression is a mediator of chronic myeloid leukaemia (CML) proliferation and CML resistance to the drug of choice for CML, the BCR/ABL inhibitor, imatinib. Together, these findings illustrate how a cancer associated tyrosine kinase can induce ROS resulting in leukaemia proliferation and drug resistance.

It has also been described that leukaemic oncogenes may also affect the transcription, stability, or activity of antioxidant proteins within leukaemic cells. For example, BCR/ABL and NF-*κ*B can increase the transcription of NRF2 and by association its regulated genes, which have been shown to have cytoprotective properties. Furthermore, activation of NRF2 requires a phosphorylation process which results in the stabilisation of NRF2 and its release from its negative regulator allowing transcription of the antioxidant genes [37]. The transcription factor NRF2 is activated by increased oxidative stress inducing protection of normal cells against electrophilic and oxidative stress [38]. This provides an example of transcriptional pathways by which leukaemic oncogenes can influence the redox environment of leukaemia cells and represent possible targets for therapeutic intervention.

## 6. NRF2 Regulated Cellular Antioxidants in Leukaemia

Our research has previously shown that current standard AML chemotherapy (cytarabine and daunorubicin) induces an increase in ROS in AML cells as part of their mechanism of cytotoxic action [39]. Furthermore, we also recently reported that malignant blasts from AML patients have inappropriate constitutive NRF2 activation, resulting in increased cell survival and chemotherapy resistance [40, 41]. The NRF2 signaling pathway is a major cellular pathway that under normal conditions protects nonmalignant cells against electrophilic and oxidative stress [38]; however, in AML as well as many other malignancies, including chronic lymphocytic leukaemia (CLL), NRF2 is constitutively activated [42]. In AML, constitutive activation of NRF2 occurs not through somatic mutation of NRF2 or its inhibitor KEAP1 but as a result of upstream constitutive activation of NF-*κ*B.

NRF2 regulates the expression of over 200 genes including many antioxidant genes and phase II enzymes such as heme oxygenase-1 (HO-1) and NAD(P)H: quinone oxidoreductase 1 (NQO1) [43, 44] and genes involved in glutathione metabolism and regeneration [45–48]. No single gene induced by NRF2 can be identified as the most important for cell protection, as cell protection is a result of the coordinated induction of NRF2 target genes. As well as the work on AML, NRF2 genes have also been dysregulated in other human blood cancers including CLL and multiple myeloma (MM). In CLL, experiments show the presence of NRF2 signaling and suggest that altered NRF2 responses may contribute to the observed selective cytotoxicity of electrophilic compounds in this disease [49]. In MM, HO-1 is increased in bortezomib-resistant MM cells, suggesting a possible role for HO-1 and NRF2 in chemotherapy resistance [50]. Together these results highlight the importance of NRF2 in human blood cancer.

### 6.1. Glutathione Metabolism, Regeneration, and Control of ROS

**Figure 1:**
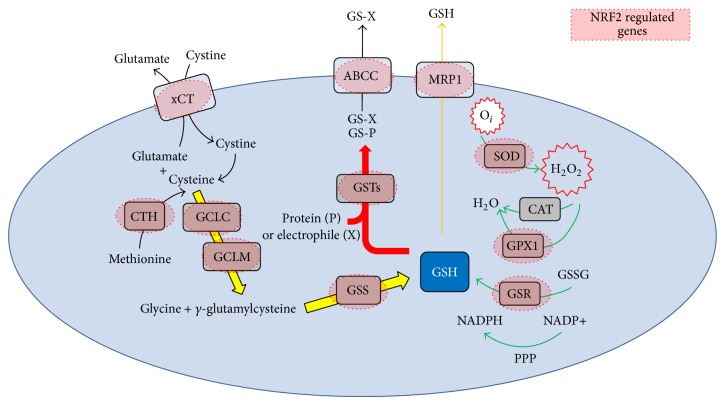
Glutathione synthesis as seen through NRF2. GSH is a two-step synthesis reaction catalysed by glutamate-cysteine ligase (GCL) and GSH synthetase. GSH is consumed in many ways, such as by oxidation or conjugation. In addition, cells may lose GSH due to export of its reduced, oxidized, or conjugated forms and intracellular GSH is regenerated via reduction at the expense of one NADPH molecule. Highlighted in red are the genes regulated by NRF2 activity.

GSH has emerged as an important regulator of chemotherapy resistance in human cancer. GSH is present in all mammalian tissues at 1–10 millimolar concentrations and protects against oxidative stress [51]. In the cell GSH exists in the thiol-reduced GSH and disulfide-oxidized (GSSG) forms [52] and its major reservoir is the cytosol (80–85%) [53–55]. GSH synthesis occurs via a two-step ATP-requiring enzymatic process and exerts a negative feedback inhibition on key rate limiting enzymes including glutathione cysteine ligase (GCL) [56, 57] either by phosphorylation or by protein expression [58]. The regulation of GSH synthesis is under tight control involving key enzymes including GCL, GSH synthetase, and GSH reductase. More importantly these enzymes are all regulated, at least in part, by NRF2 through its activation of the ARE [59]. This highlights the importance of addressing the link between NRF2 and GSH in disease, especially leukaemia.  Figure 1 shows the link between NRF2 and GSH synthesis and regeneration.

It is becoming apparent that NRF2 is the main transcription factor that controls the regulation of many aspects of GSH synthesis and regeneration [60, 61]. Importantly the regulation of GCL at the transcriptional level is essential for the maintenance of GSH homeostasis in response to oxidative stress. In addition, levels of GCLC and GCLM are decreased in NRF2 knockout mice; the resulting lack of GSH synthesis is lethal during embryogenesis [62]. Moreover, GSH synthetase which catalyses the second step of GSH generation is also regulated by NRF2 and overexpression of either NRF1 or NRF2 induced the GSS promoter activity by 130 and 168%, respectively. Other genes involved in GSH metabolism, regeneration, and function are also regulated by NRF2 activation, which include GSH S-transferases (GSTs), GSH reductase (GR), and GSH peroxidase (GPX) [48, 63, 64]. Together, this information suggests that NRF2 controls the effectiveness of GSH to combat the excess of ROS.

Hydrogen peroxide is one of the main activators of the NRF2-KEAP1 pathway. It is metabolised by GPX in the cytosol resulting in GSH being oxidized to GSSG in the mitochondria. GSSG can be reduced back to GSH by GR at the expense of NADPH, thereby forming a redox cycle, where organic peroxides can be reduced by either GPX or GSH S-transferase (GST) [65]. GSTs are a family of phase II conjugation enzymes under the regulation of the NRF2/ARE pathway [66]. The main role of GST is to catalyse the detoxification of various harmful compounds [67]. This detoxification process is under the tight control of NRF2 as GST mRNA and protein expression are decreased in NRF2-null mice, and NRF2 is required for GST induction [68]. Moreover, the mRNA expression of GST is markedly increased in KEAP1-null mice [69]. This provides evidence that not only GSTs but also many other enzymes that are involved in GSH synthesis and regeneration are coordinately regulated by NRF2 and justifies the necessity to address the NRF2 GSH axis in human cancers, especially leukaemia.

### 6.2. NRF2, GSH, and Leukaemic Cell Survival

Although NRF2 is protective against tumorigenesis by reducing the amount of ROS and DNA damage in cells, tumour cells were found to be capable of harnessing the protective function of NRF2 for their own protection and survival [42, 70]. Indeed, NRF2 activity itself is elevated in some leukaemia types where it contributes to leukaemogenesis [71]. Elevated nuclear localization of NRF2 and the subsequent genetic changes result in reduced sensitivity to proteasome inhibitors in AML cell lines [41], suggesting that NRF2 may also regulate sensitivity to ROS-producing therapeutic agents. Moreover, molecular analyses have revealed that treatment with stress inducers (e.g., tumour necrosis factor) results in increased NRF2 activity in THP-1, HL-60, and AML 193 cell lines, which in turn increases the transcription of antioxidants [72].

Primitive hematopoietic stem and progenitor cells reside within the bone marrow and express the CD34 surface antigen [73, 74]. Moreover, primitive AML cells also generally express CD34 and are more resistant to chemotherapy [74, 75]. A recent study by Pei et al. evaluates the characteristics of primary CD34+ cells derived from patients with AML in comparison to normal CD34+ controls [76]. This is consistent with the finding that CLL cells have elevated levels of reactive oxygen species (ROS) compared to normal controls [77]. Taken together, this suggests that altered GSH content might be a common property of primary hematopoietic malignant tissues.

The prognostic value of GST deletions in adult AML, including individuals with GSTM1 or GSTT1 deletions (or deletions of both), is found to have enhanced resistance to chemotherapy, lower complete remission, and a shorter survival [78]; this further supports the suggestion of a disturbed glutathione metabolism in AML cells. AML cells have elevated expression of multiple GSH metabolising enzymes including GCL and GST compared to control CD34+ cells and knockdown of GCLC or GPX1 impaired the growth of leukaemic cells in vitro [76]. Moreover, a significantly decreased GSH to GSSG ratio further indicates aberrant glutathione homeostasis in AML cells; this is consistent with findings of increased basal levels of nuclear NRF2 in primary AMLs [41]. This highly suggests that increased NRF2 activity in AML cells is responsible for the elevated expression of these genes as a mechanism by which AML cells compensate for increased oxidative stress in leukaemic cells. The aberrant glutathione metabolism presents a unique and potentially useful asset for targeting of primitive leukaemic cells.

## 7. Conclusion

ROS play an important functional role in human leukaemia. NRF2 and its control of GSH regulate ROS. Recent data suggests that GSH is fundamental to NRF2 function in AML suggesting that this pathway may yield future therapeutic targets for leukaemia cells in which GSH is dysregulated.

## Abbreviations

ABCC/MRP1: Multidrug resistance proteins
AML: Acute myeloid leukaemia
GSR: GSH reductase
GPX1: GSH peroxidase
GST: GSH S-transferase
NADPH: Nicotinamide adenine dinucleotide phosphate
ATP: Adenosine triphosphate
ADP: Adenosine diphosphate
H_2_O_2_: Hydrogen peroxide
GCL: Glutamate-cysteine ligase
ROS: Reactive oxygen species
NRF2: Nuclear factor (erythroid-derived 2)-like 2
FR: Free radicals
RNS: Reactive nitrogen species
CML: Chronic myeloid leukaemia.
